# Notes on Organization

**Published:** 1896-12

**Authors:** 


### NOTES ON ORGANIZATION.

The Brazos Valley Medical Society.—It is particularly gratifying to the Journal to note the growth of this new society; and of organization, generally, throughout the State. To this the Texas Medical Journal has devoted its best energies for years: organization is—and has been its key-note and mission, for it was early recognized that "in union there is strength," and that only a united profession can make itself felt.

The profession of Texas numbers amongst its members men, the peers of any in the United States in point of professional attainments. We have, indeed, many really able and influential men in the profession; but the past has sufficiently demonstrated that the very best directed efforts of some of the ablest men in the profession have been powerless to impress upon the legislature that the medical profession have rights that ought to be respected. They have never been impressed with the strength and dignity of the medical profession as an element of population. In brief—our efforts at medical legislation have had too much of an individual character, and have been spasmodic. The legislators have never been made to feel that the request for a rational medical act: one limiting the privileges of the medical profession to the qualified—has been the voice of the profession. We are not united;—and until we are, the profession will never wield that influence which its dignity and learning—to say nothing of numbers—entitle it to.

The Journal, for the reason of its original and persistent advocacy of thorough organization—has been recognized by most of the local organizations as the leading spirit, and has been, by unanimous vote, in most instances—made the official organ or mouth-piece of each organized society. We note with interest in the proceedings of the Brazos Valley Society that they have a plan to propose at the next meeting of the State Society for perfecting local organizations and enrolling all local societies in the membership of the State Association. Many plans have been proposed. The JOURNAL has insisted, time and again, that the best way, if not the only one—is to make the meetings attractive, interesting; to arouse professional pride, and a spirit of emulation; and this plan has worked well—so far; the Brazos Valley, and many other newly-organized societies testifying to its efficiency. The meetings are interesting; and it is particularly gratifying to see so much pride and interest taken in them. If the Brazos Valley Medical Association has a better plan to propose, by all means let us have it.

There is something wrong,—somewhere. Of a profession numbering at least four thousand,—by many said to number five thousand,—it is a reproach to our intelligence, or our enterprize or our spirit—that only about eight or ten per cent, say four hundred—should be in full fellowship in the State Association.

A new era is dawning. All over the State, meetings are being called and held;—and when the profession has formed itself into local bodies—let the members, then, by virtue of local membership—become members of the State Association; remove all the obstacles that it is possible to remove from the doors of the State Association; guard well the portals *at home*, and let home-membership be taken as evidence of fitness for State membership, and the principal obstacle—next to the still unnecessarily large "dues"—will have been removed. Under existing rules —an applicant for membership must attend,—however distant, carrying his diploma with him; or if he make application for membership—*in absentia*—through some friend—he has to send his diploma and a five dollar bill by express. All will agree that one must be quite anxious for membership to do this.

The JOURNAL notes with satisfaction a call for organization at Houston December 9th, of the Southern Texas Medical Association. We will look forward to that event with interest, and meantime we urge all who are in that district to make it a point to attend.

The Galveston County Medical Society, we much regret to see, has lost its grip, and passed into a dormant state. Secretary West, of the State Association, residing at Galveston—ought, it seems to us, to be able to arouse, or re-arouse, his colleagues to a sense of the importance of keeping step to the music in the march of organization.

At San Antonio, as will be seen by reading President Hadra's retiring address—the Southwest Texas Medical Association has lost none of its old-time vim and interest. We commend this address to our readers as being paying, profitable, and most interesting reading. Dr. Hadra has the humor of a Lamb—if not the innocence of one, the wit of a Jerrold, and his satire is worthy of Juvenal or Dickens. The doctor tells some truths that are not very flattering to our vanity.

The Terrell Medical Society has not been heard from in some time. Dr. F. S. White is now the secretary. While Dr. Orr was secretary the JOURNAL was favored with proceedings and papers. Dr. White must rally his colleagues, if he and they would not get left. The Williamson, Burnet and Milam Society has not been heard from lately, either.

We know, it has been a hard year with the doctors; but now —when, by common consent, an era of prosperity is about to set in—let all forget the hardships and disappointments of the past, and set forward with renewed energy and vigor, each one determined to do what he can for the advancement and interest of his profession; and he can in no way better do that than by actively aiding in organizing the profession in his section, or if it has relapsed into desuetude—by rallying the members to one more effort; and when the State Medical Association meets next April, let there be delegates there, representing an organization in every county in the State!

Then—and not till then,—when the State Association shall have become, really, and numerically, representative of the profession of the State—will members of the legislature harken to the appeals for medical legislation so much needed to put Texas abreast of her sister States in medical and sanitary matters, and to put an end to quackery, now so rampant throughout the land.

